# Structure-function analysis indicates that sumoylation modulates DNA-binding activity of STAT1

**DOI:** 10.1186/1471-2091-13-20

**Published:** 2012-10-08

**Authors:** Juha Grönholm, Sari Vanhatupa, Daniela Ungureanu, Jouni Väliaho, Tuomo Laitinen, Jarkko Valjakka, Olli Silvennoinen

**Affiliations:** 1 Institute of Biomedical Technology, University of Tampere, Biokatu 8, Tampere, FIN-33014, Finland; 2 Faculty of Health Sciences, School of Pharmacy, University of Eastern Finland, Kuopio, FIN-70211, Finland; 3 Department of Internal Medicine, Tampere University Hospital, Tampere, FIN-33521, Finland

**Keywords:** Signal transduction, Transcription factors, Sumoylation, Signal transducers and activators of transcription (STATs), Interferon

## Abstract

**Background:**

STAT1 is an essential transcription factor for interferon-γ-mediated gene responses. A distinct sumoylation consensus site (ψKxE) ^702^IKTE^705^ is localized in the C-terminal region of STAT1, where Lys703 is a target for PIAS-induced SUMO modification. Several studies indicate that sumoylation has an inhibitory role on STAT1-mediated gene expression but the molecular mechanisms are not fully understood.

**Results:**

Here, we have performed a structural and functional analysis of sumoylation in STAT1. We show that deconjugation of SUMO by SENP1 enhances the transcriptional activity of STAT1, confirming a negative regulatory effect of sumoylation on STAT1 activity. Inspection of molecular model indicated that consensus site is well exposed to SUMO-conjugation in STAT1 homodimer and that the conjugated SUMO moiety is directed towards DNA, thus able to form a sterical hindrance affecting promoter binding of dimeric STAT1. In addition, oligoprecipitation experiments indicated that sumoylation deficient STAT1 E705Q mutant has higher DNA-binding activity on STAT1 responsive gene promoters than wild-type STAT1. Furthermore, sumoylation deficient STAT1 E705Q mutant displayed enhanced histone H4 acetylation on interferon-γ-responsive promoter compared to wild-type STAT1.

**Conclusions:**

Our results suggest that sumoylation participates in regulation of STAT1 responses by modulating DNA-binding properties of STAT1**.**

## Background

STAT1 (Signal transducer and activator of transcription 1) is the founder member of the STAT family of transcription factors and plays a critical role in interferon (IFN) regulated gene responses. IFN-γ activates STAT1 through Janus kinase (JAK)-mediated phosphorylation of Tyr701. Activated STAT1 homodimerizes and translocates to the nucleus where it binds to DNA and initiates transcription of IFN-γ-regulated genes [1,2]. The X-ray structure (1bf5.pdb) of the DNA-bound STAT1 dimer shows a contiguous C-shaped clamp around DNA, that is mediated by specific interactions between the SH2 domain and the tyrosine (Tyr701) phosphorylated C-terminal tail segment (residues 700–708) of the monomers [3].

Small ubiquitin-like modifier (SUMO) proteins belong to the family of ubiquitin-like protein modifiers, collectively termed Ubls, that are covalently attached to substrate proteins by a cascade of enzymatic reactions [4]. The conjugation is regulated by distinct SUMO specific enzymes such as E1 activating enzyme Aos1/Uba2 and the E2 conjugase Ubc9. The protein inhibitor of activated STAT (PIAS) family of proteins, PIAS1, PIAS3, PIASx and PIASy have been shown to function as E3-type ligases to promote SUMO conjugation to the target proteins [5-7]. PIAS1 functions as a negative regulator of STAT1-mediated transcription through interaction with the dimerized STAT1 and by inhibiting STAT1 DNA-binding [8,9]. Interestingly, PIAS proteins have also been shown to promote sumoylation of STAT1 at single Lys703 amino acid residue within the SUMO consensus sequence (ψKxE, where ψ indicates large hydrophobic amino acid and x refers to any amino acid) ^702^IKTE^705^ in the C-terminal region of STAT1 [10,11]. Furthermore, it has been shown that mitogen activated protein kinase (MAPK)-induced phosphorylation of Ser727 in STAT1 promotes interaction of STAT1 with PIAS1 and leads to enhanced STAT1 sumoylation [12].

Several studies suggest that sumoylation has a negative effect on STAT1-mediated gene responses [11,13,14]. Sumoylation site Lys703 is in a close proximity to Tyr701 that is required for STAT1 activation, and sumoylation has been shown to directly inhibit STAT1 Tyr701 phosphorylation [15-17]. Sumoylation has also been shown to prevent condensation of STAT1 oligomers in the nuclear paracrystals, and thereby increase the solubility of STAT1 and promote its dephosphorylation [17]. Recently, it was discovered that in addition to human STAT1, also murine STAT5 and *Drosophila* Stat92E are regulated through SUMO conjugation, confirming that sumoylation has an evolutionary conserved role in regulation of the cytokine signaling [18,19].

This study was aimed to investigate the mechanism by which sumoylation regulates STAT1 activity. Inspection of molecular model indicates that SUMO consensus site is well exposed in STAT1 dimer, and it is accessible for propitious interactions with regulatory proteins. The constructed molecular model of SUMO-1 conjugated STAT1 dimer further suggested that SUMO-1 moiety is oriented towards DNA, thus able to affect the DNA-binding properties of STAT1 with its presence. The molecular model was supported by experimental evidence, and oligoprecipitation experiments indicated that sumoylation deficient STAT1 E705Q mutant display higher DNA-binding activity on STAT1 target gene promoters when compared to STAT1 wild-type (WT). Furthermore, sumoylation deficient STAT1 mutant showed enhanced histone H4 acetylation at the *Gbp-1* promoter.

## Methods

### Plasmids

STAT1 WT-HA, STAT1 WT-Flag, STAT1 K703R-HA, STAT1 E705A-HA and STAT1 Y701F plasmids were previously described [11-13]. STAT1 E705Q mutation was created with site directed polymerase chain reaction (PCR) mutagenesis using primers: 5’-GGAACTGGATATATCAAGACTCAGTTGATTTCTGTGTC-3’ and 5’-GACACAGAAATCAACTGAGTCTTGATATATCCAGTTCC-3’.

pSG5-SUMO-1-His was provided by Dr. A. Dejean [20]. The GAS-luciferase construct contains the IFN-γ regulated GAS element [21]. Flag-tagged SENP1 and SENP1 C603S were previously described [22].

### Cell culture

Human HeLa cells and monkey Cos-7 cells were cultured in Dulbecco’s modified Eagle’s medium (DMEM) supplemented with 10% fetal bovine serum (FBS) (Gibco), 100 U/ml penicillin and 50 mg/ml streptomycin. Human fibrosarcoma U3A cells (kindly provided by Dr. I. Kerr) were cultured in DMEM supplemented with 10% Cosmic calf serum (CCS) (HyClone, Logan, UT) and 100 U/ml penicillin and 50 mg/ml streptomycin. Stable U3A-STAT1 WT-HA and U3A-STAT1 K703R-HA clones were previously described [11,13].

### Reporter gene assays

Approximately 0.2 × 10^6^ HeLa cells were plated on to 24-well plates and transfected with 0.25 μg CMV-β-galactosidase reporter plasmid as an internal transfection efficiency control and 0.25 μg GAS-luciferase construct together with empty pcDNA3.1 vector as a control or with increasing amount of SENP1-Flag or SENP1 C603S-Flag. After 36 hours the cells were serum starved over night with 0.5% FBS in DMEM, following stimulation with 100 ng/ml human IFN-γ (Immugenex, Los Angeles, CA) for additional 6 hours and lysed in Promega’s reporter lysis buffer according to the manufacturer’s instructions. Luciferase activity was measured using Luminoscan Ascent (ThermoElectron Corporation, Finland) and normalized against β-galactosidase activity of the lysates.

### Immunoprecipitation, co-immunoprecipitation and Immunoblotting

Total amount of 3 × 10^6^ Cos-7 cells were transfected with 2 μg of STAT1-WT, 1 μg of SUMO-1, together with 2 μg of SENP1 or 2 μg of SENP1 C603S mutant. The cells were lysed in Triton X lysis buffer (50 mM Tris–HCl, pH 7.5, 150 mM NaCl, 1 mM EDTA, 50 mM NaF, 10% glycerol, 1% Triton X-100) supplemented with protease inhibitors including 10 mM NEM (Sigma Aldrich, St. Louis, MO). The lysates were incubated with anti-STAT1 antibody (monoclonal anti-STAT1 N-terminus, Transduction Laboratories, BD Biosciences, Franklin Lakes, NJ) and the immunocomplex was washed and subjected to SDS-PAGE electrophoresis. STAT1 and sumoylated STAT1 protein levels were determined by using anti-STAT1 (Transduction Laboratories) and anti-SUMO-1 (Zymed) antibodies. SENP1 protein levels from the whole cell lysates were determined by immunoblotting with anti-Flag antibody (Sigma-Aldrich, St Louis, MO). For co-immunoprecipitation experiments 1.6 × 10^6^ Cos-7 cells were transiently transfected with 3 μg of STAT1-HA and 3 μg of STAT1-Flag with or without 4 μg of SUMO-1-His using L-PEI transfection reagent as described [23]. After 48 hours cells were lysed in buffer containing 20 mM HEPES pH 8.0, 100 mM NaCl, 1% Triton X-100, 10% glycerol, 50 mM NaF and 1mM EDTA supplemented with 10 mM NEM and protease inhibitors. Equal amounts of whole cell lysates were incubated for 3 hours in rotator at 4°C in the presence of 20 μl of anti-Flag M2 agarose beads (Sigma Aldrich, St. Louis, MO). The beads were washed 3 times with the lysis buffer and anti-Flag immunoprecipitated proteins were released from the beads by incubating them in the presence of Flag-peptide (F3290, Sigma Aldrich, St. Louis, MO) for 30 min at 4°C. Proteins were separated by SDS-PAGE and STAT1 was detected by immunoblotting using anti-HA antibody (clone 16B12, Covance, Princeton, NY). STAT1 and SENP1 protein levels from luciferase assay samples were analysed by immunoblotting using anti-STAT1 and anti-Flag antibodies, respectively.

### Oligoprecipitation

Total amount of 5 × 10^5^ U3A cells were transfected with 6 μg of STAT1 WT-HA or STAT1 E705Q-HA or STAT1 Y701F-HA mutants together with 4 μg of SUMO-1-His using L-PEI transfection reagent. After 48 hour incubation at 37°C cells were either left unstimulated or stimulated with 100 ng/ml of human IFN-γ (Immugenex, Los Angeles, CA) for total of 1 hour and by osmotic shock (0.3 M sorbitol) for 15 minutes. The cells were lysed in lysis buffer (0.5% Triton X-100, 100 mM Tris–HCl pH 7.5, 0.2 mM EDTA, 300 mM NaCl, 1 mM DTT, 10% glycerol, 1 mM NaF) supplemented with protease inhibitors. The lysates were diluted fourfold with dilution buffer lacking NaCl (0.1% Triton X-100, 100 mM Tris–HCl pH 7.5, 0.2 mM EDTA, 1 mM DTT, 1.0% glycerol, 1 mM NaF supplemented with protease inhibitors).

For the binding assay, a biotinylated oligonucleotide containing the GAS from the human *Gbp-1* gene (5’-GGATCCTACTTTCAGTTTCATATTACTCTAAAT-3’) or STAT1 binding site from human *Irf-*1 gene (5’-GGATCCCAACAGCTTGATTTCCCCGAAATGACGGCA-3’) promoter was annealed and 3 nmols of biotinylated oligonucleotide duplex were rotated for 2 hours at 4°C with Neutravidin agarose to form GAS-agarose affinity beads. Diluted cell extracts were precleared with Neutravidin beads and then incubated with GAS-agarose affinity beads for 2 hours in rotator at 4°C. The beads were then washed four times with buffer containing 0,2% Triton X-100, 10 mM HEPES pH 7.9, 2 mM EDTA, 1 mM EGTA, 150 mM KCl, 10% glycerol and 1 mM NaF. GAS-agarose affinity bead-bound proteins were subjected to SDS-PAGE and detected by immunoblotting with phospho-tyrosine (Tyr701)-specific STAT1 antibody (Cell Signaling). The Western blot membranes were stripped and reprobed with anti-HA antibody (Covance, Princeton, NY) to detect total amount of DNA-bound STAT1. Detected bands were quantified by using ImageJ image analysis software and analyzed after background subtraction.

### Chromatin immunoprecipitation

Stable U3A-STAT1 WT-HA and U3A-STAT1 K703R-HA clones were starved and left unstimulated or stimulated with IFN-γ for 1h. Chromatin immunoprecipitation (ChIP) was performed as previously described [24] using anti-acetyl-histone H4 antibody (Upstate Biotech) or as a control, anti-IgG antibody (Santa Cruz Biotechnology). DNA was analyzed for human *Gbp-1* promoter by quantitative-RT-PCR with the following primers: 5’-AGCTTCTGGTTGAGAAATCTTT-3’ and 5’-CCCTGGACTAATATTTCACTG-3’. Quantitative-PCR was done using SYBR green I kit (Qiagen) according to manufacturer’s instructions. The values from ChIP assays were normalized to the total input DNA.

### Molecular modeling

A 3D-structure of STAT1 dimer with DNA has been built using crystal structure of tyrosine phosphorylated STAT1-DNA complex (PDB code:1BF5) [3]. The molecular geometry of the loop 684–699 in the SH2 domain was calculated using the program Sybyl with Amber 7 FF99 force field parameters. The initial model for the loop region was constructed using the crossover loop structure from the SUMO-1-TDG (PDB code:1wyw) [25] as a template. First, during the energy and geometry minimization for the loop all hydrogen atoms and non-constraints were included in the protocol. Second, during the molecular dynamic refinement the constraints were on for outer part of the loop in the SH2 domain. After the loop modeling we used the deposited coordinates of SUMO-1 (PDB code:1wyw) [25] in our model. The SUMO-1 was set nearby the constructed loop 684–699 so that its C-terminal residue (Gly97) is in the vicinity of the Lys703 of the STAT1 and the loop can form a new β-strand to an existing antiparallel β- sheet structure in the SUMO-1. The loop 684–699 was also modeled with InsightII (2005, Accelrys Inc. InsightII - molecular modeling program. http://www.accelrys.comomain; 1wyw.pdb). The entire structure was then subjected to energy minimization using the molecular mechanics force field CVFF (consistent valence force field) and the steepest descent algorithm implemented under Insight II Discover program. During the minimization, the DNA and the atoms of the STAT1 residues 136–686 and 700–710 were fixed.

## Results

### SENP1 deconjugates SUMO-1 from STAT1 and enhances STAT1-mediated gene expression

**Figure 1:**
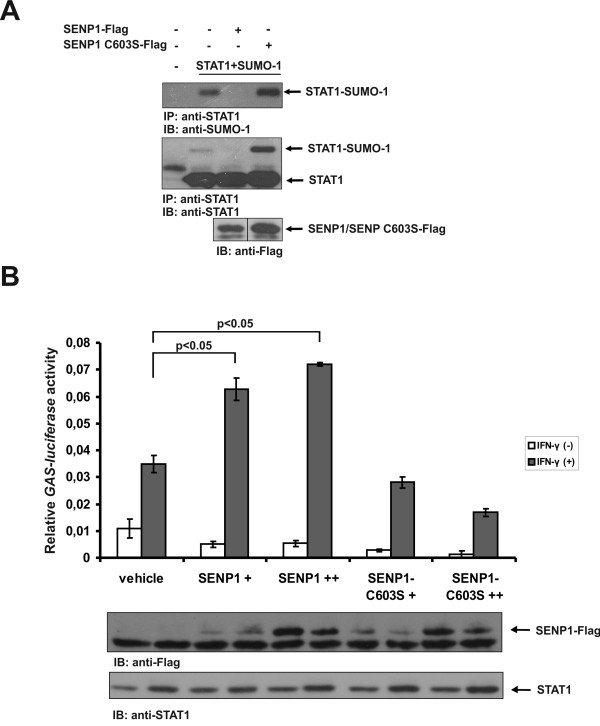
**Sumoylated STAT1 is target for SENP1-mediated desumoylation.** (**A**) SENP1 desumoylates STAT1 in Cos-7 cells. Cos-7 cells were transiently transfected with expression vectors containing STAT1-HA, SUMO-1-His and Flag-tagged SENP1 or SENP1 C603S mutant as indicated. After 36 hours the cells were lysed and total cell lysates were immunoprecipitated with anti-STAT1 antibody and immunoblotted with anti-SUMO-1 and anti-STAT1 antibodies. SENP1 protein levels were verified by immunoblotting with anti-Flag antibody. (**B**) SENP1 overexpression augments STAT1 transcriptional activity in cultured cells. HeLa cells were transfected with either empty vector or with increasing amount of Flag-tagged WT SENP1 or SENP1 C603S together with GAS-luciferase reporter and CMV-β-galactosidase reporter for normalizing transfection efficiency. After 36 hours cells were starved over night and stimulated with 100 ng/ml of human IFN-γ for 6 hours prior to cell lysis. Luciferase values were normalized against β-galactosidase values. SENP1 and endogenous STAT1 expression levels were verified from the lysates by immunoblotting using anti-Flag and anti-STAT1 antibodies, respectively. Error bars indicate SD from 3 independent transfections. IB: immunoblot, IP: immunoprecipitation.

The covalent modification of proteins by SUMO-1 is known to be a reversible process [22]*.* We wanted to confirm the effect of sumoylation of STAT1 in cells by analyzing whether STAT1 is a substrate for SUMO protease SENP1. For this, STAT1, SUMO-1 and SENP1-Flag or catalytically inactive SENP1 C603S-Flag were transfected into Cos-7 cells and equal amounts of protein was immunoprecipitated with anti-STAT1 antibody and immunoblotted with anti-STAT1 and anti-SUMO-1 antibodies. As shown in Figure 1A, co-transfection of SENP1 WT completely abolished the slower migrating STAT1-SUMO-1 band indicating that sumoylation of STAT1 is reversible and SENP1 can act as a SUMO-specific isopeptidase for STAT1.

The identification of STAT1 as a substrate for SENP1 prompted us to investigate whether SENP1-mediated desumoylation affects the transcriptional activity of STAT1. For this purpose, we analysed the activity of STAT1-responsive GAS-luciferace reporter in HeLa cells transfected with different concentrations of SENP1 WT or SENP1 C603S mutant. As shown in Figure 1B, overexpression of SENP1 significantly increased the transcriptional activity of endogenous STAT1. In contrast, overexpression of catalytically inactive SENP1 C603S resulted in a dose-dependent decrease in GAS-luciferase activity, most probably by blocking the interaction of endogenous SENP1 with STAT1, leading to increased level of SUMO-modified STAT1 in the cells. This effect is also seen in Figure 1A, where STAT1 sumoylation is significantly increased when SENP1 C603S is co-transfected into the Cos-7 cells. Collectively, these results indicate that desumoylation of STAT1 enhances its transcriptional activity. These results are in line with previously reported results that sumoylation deficient STAT1 mutants display higher transcriptional activity at STAT1 target gene promoters [13,14].

### Molecular model of the SUMO conjugated STAT1 dimer

**Figure 2:**
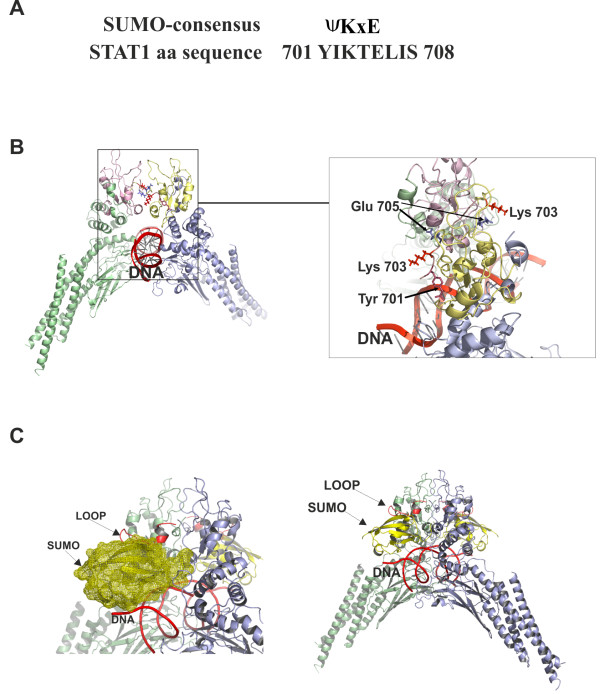
**Ribbon diagram of the STAT1 homodimer-DNA complex (PDB code:1bf5) and model of SUMO conjugated STAT1 dimer.** (**A**) SUMO consensus element and amino acid (aa) sequence of SUMO-conjugation site in STAT1. The symbol ψ indicates large hydrophobic amino acid and x can be any amino acid. (**B**) Coiled-coil domain, DNA-binding domain and linker region of one monomer are colored green and the SH2 domain is colored in salmon red. SH2 domain of adjacent monomer is colored light yellow and other domains are blue. DNA strand is labelled and colored in red. Residues, pTyr701 (salmon red), Lys703 (red) and Glu705 (blue) are shown in a stick representation. All ribbon diagrams presented in Figure 2 are colored uniformly and produced using PyMOL (http://www.pymol.org). (**C**) The model of SUMO conjugated STAT1 was constructed by superimposing the X-ray structures of SUMO-1 from the complex of SUMO-1-TDG (PDB code:1wyw) and the structure of DNA bound STAT1 dimer (PDB code:1bf5). The molecular geometry of the loop 684–699 in the SH2 domain was calculated using the program Sybyl with Amber 7 FF99 force field parameters. Green and blue colours were used to distinguish monomers in a ribbon diagram of DNA bound STAT1 dimer. The loop and DNA are labeled and colored with red. The SUMO-1 was incorporated between the loop and DNA in conjugation distance on both sides of dimer. SUMO-1 is represented in ribbon and its molecular surface in yellow. Graphics were produced using PyMOL (http://www.pymol.org).

IFN-γ-induced activation of STAT1 transcription factor requires phosphorylation of Tyr701 leading to its rapid homodimerization followed by translocation to the nucleus and binding to the target gene promoters [3]. To obtain further insight on the mechanisms and consequences of SUMO conjugation to STAT1, we modeled the SUMO moiety and analysed its orientation in STAT1 dimer using information from the published structure of Tyr701 phosphorylated STAT1 homodimer bound to DNA [3]. The interaction between STAT1 monomers is formed between SH2 domain and phosphorylated tyrosine 701 of an adjacent monomer, so that the phosphate group is recognized by the strictly conserved Arg602 residue that rises up from the interior of the SH2 domain. Analysis of the SUMO conjugation site (Figure 2A) demonstrated that the side chains of Lys703 of both monomers formed a projection on the same orientation with DNA, and formed a suitable site for covalent isopeptide bond between STAT1 and SUMO (Figure 2B). Monomeric STAT1 molecules have a tendency to form dimers in solution due to the β-sheet structure between two C-tail segments of STAT1. The formation of the β-sheet structure is mediated by hydrogen bonding through the backbone atoms of Glu705, Ile707 and Val709. The formed dimer structure is further stabilized by interactions in the hydrophobic core between SH2 domains [3]. Substitution of Lys703 with Arg, a commonly used sumoylation abrogating mutation, or substitution of Glu705 with Gln, are not predicted to interfere phosphorylation of Tyr701 or interrupt interactions involved in the dimerization interface, or directly affect DNA-binding properties of STAT1 (Additional file 1: Figure S1 and Additional file 2: Figure S2).

The crystal structure of thymine DNA glycosylase (TDG) conjugated to SUMO-1 has revealed that TDG forms two dissimilar molecular interfaces with SUMO-1. The covalent contact to SUMO-1 occurs at the Lys330 residue, but another interface is a β-sheet structure formed by β-strands of TDG and SUMO-1 [25]. The structure of STAT1 dimer (PDB code: 1bf5) has a linker region (aa 684–699; loop structure) that is invisible in the electron density maps [3]. The immediate vicinity of sumoylation site to residues in both ‘ends’ of the loop structure pointed us to investigate and remodel this loop. To get insight on this, we constructed a model of sumoylated STAT1 dimer using previously published coordinates of conjugated SUMO-1 [25]. The loop amino acids 684–699 was reconstructed using two programs Sybyl with Amber7 FF99 force field (Figure 2C) and InsightII, and the analysis resulted in two highly similar loop models. SUMO-1 was positioned on conjugation distance, and the constructed loop structure was presented adjacent to β-sheet structure of SUMO-1. This model proposes that interface between SUMO-1 and the loop structure of STAT1 can direct the SUMO-1 moiety towards DNA, creating a steric hindrance that can affect DNA-binding of sumoylated STAT1 dimer.

### Sumoylation deficient STAT1 shows increased DNA-binding activity

The molecular model suggested that sumoylation may alter the DNA-binding properties of STAT1. Mutation of Lys703 (K703R) or Glu705 (E705A) within the sumoylation consensus sequence in STAT1 abolish sumoylation of STAT1 and leads to enhanced STAT1 transcriptional activity [11,13]. Thus, we wanted to investigate if the DNA-binding activity of sumoylation deficient STAT1 mutants differ from the DNA-binding properties of the WT STAT1. Amino acids essential to SUMO conjugation reside in the close proximity of the STAT1 activating Tyr701 phosphorylation site and therefore the mutations in the sumoylation site may affect to the tyrosine phosphorylation or dephosphorylation properties of STAT1. E705Q mutation in STAT1 is predicted to have minimal structural consequences to STAT1 but it abolishes STAT1 sumoylation (Additional file 2: Figure S2) [17]. To analyse the phosphorylation of different sumoylation deficient STAT1 mutants, U3A cells lacking endogenous STAT1 were transfected either with STAT1 WT or with sumoylation deficient K703R, E705A or E705Q mutants. Phosphorylation deficient STAT1 Y701F mutant was used as a negative control. After IFN-γ stimulation cells were lysed and equal amounts of protein were separated in SDS-PAGE and Tyr701 phosphorylation was analyzed by using phospho-Tyr701-STAT1 specific antibody (anti-pSTAT1, Rabbit polyclonal antibody, Cell Signaling). The pSTAT1 antibody detected the Tyr701 phosphorylation of STAT1 E705Q, while the STAT1 E705A showed only a weak signal and the antibody failed to detect IFN-γ stimulated STAT1 K703R (Additional file 3: Figure S3A). The difference is likely to be caused by the altered amino acid sequence within or in the proximity of the epitope for the pSTAT1 antibody since another pSTAT1 antibody (New England Biolabs) readily detected also K703R and E705A mutants after pervanadate stimulation (data not shown). The SUMO deficient STAT1 mutant E705Q was chosen for further DNA-binding studies.

**Figure 3:**
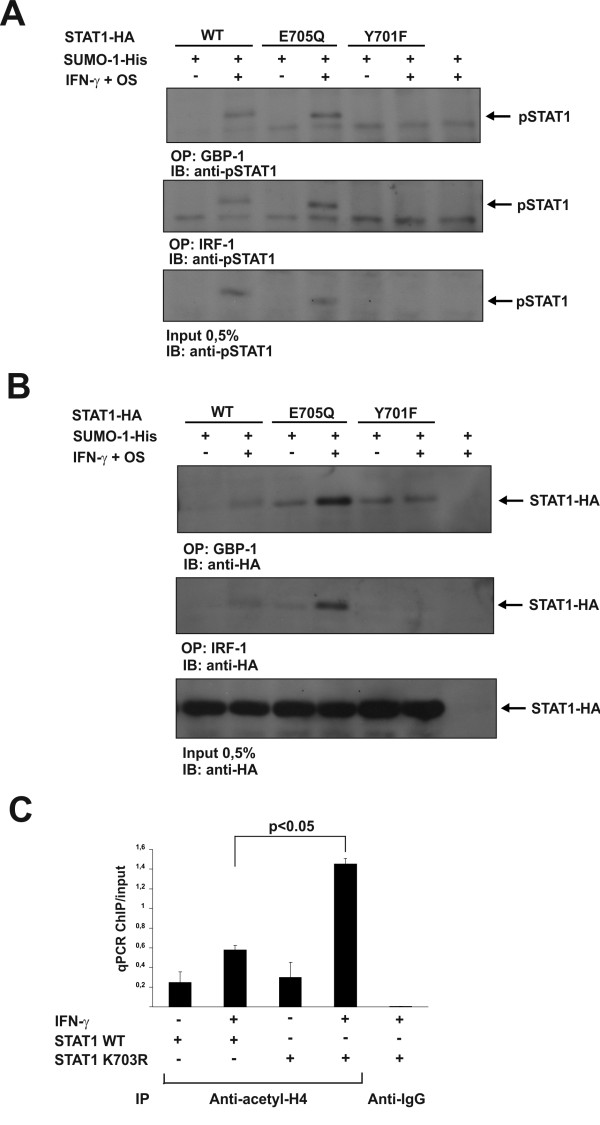
**Sumoylation deficient STAT1 mutant shows enhanced DNA-binding on STAT1 dependent promoters and enhanced histone H4 acetylation at*Gbp-1*promoter.** (**A**) The binding of phosphorylated STAT1 WT, E705Q and Y701F to *Gbp-1*- and *Irf-1*-oligos. Plasmids were transfected to U3A cells as indicated. Cells were either stimulated or left unstimulated with IFN-γ for 1h and osmotic shock (OS) for 15 minutes to induce STAT1 Tyr701 phosphorylation and STAT1 sumoylation, respectively. Equal amounts of total cell lysates were subjected to oligoprecipitation with *Gbp-1*- and *Irf-1*-oligos following immunodetection with pSTAT1 antibody (upper and middle panel, respectively). Tyr701 phosphorylated STAT1 WT and E705Q from the whole cell lysates (0.5% input) were blotted using anti-pSTAT1 antibody (lower panel). (**B**) The Western blot membranes from 3A were stripped and reprobed with anti-HA antibody to detect DNA bound STAT1 and the expression level of STAT1s in the lysates. (**C**) Sumoylation defective STAT1 K703R shows enhanced acetylation of histone H4 at the IFN-γ-dependent *Gbp-1* promoter. Stably transfected STAT1 WT and STAT1 K703R U3A clones were starved overnight and stimulated as indicated. Immunoprecipitation of cross-linked chromatin was performed with anti-acetyl H4 antibody and anti rabbit IgG as a control antibody. DNA was extracted and analysed by qPCR using primers specific for IFN-γ inducible *Gbp-1* promoter sequence. The values from immunoprecipitation were normalized to the total input DNA. The mean qPCR values ± SD from three independent experiments are shown. IB: immunoblot, IP: immunoprecipitation, OP: oligoprecipitation, OS: osmotic shock.

In order to study the DNA-binding properties of STAT1, we performed oligoprecipitation experiments using two biotinylated oligos, one containing STAT1-binding site from *Gbp-1* promoter and another with STAT1-binding site from *Irf-1* gene promoter. U3A cells were transfected either with HA-tagged STAT1 WT or STAT1 E705Q or STAT1 Y701F mutants together with His-tagged SUMO-1. Cells were left unstimulated or treated with IFN-γ and osmotic shock to induce STAT1 Tyr701 phosphorylation and STAT1 sumoylation, respectively [12]. STAT1 WT and the STAT1 mutants were oligoprecipitated from the whole cell lysates and the phosphorylation of STAT1 was detected with anti-pSTAT1 antibody. Sumoylation deficient STAT1 E705Q showed increased binding to both oligos when compared to STAT1 WT (Figure 3A upper and the middle panel). In the lysates the E705Q mutant was slightly less tyrosine phosphorylated than STAT1 WT (Figure 3A, lower panel), indicating that the increased DNA-binding of STAT1 E705Q is not a consequence of its altered Tyr701 phosphorylation. Restaining the membranes from Figure 3A with anti-HA antibody also showed that more STAT1 E705Q was bound to DNA when compared to STAT1 WT (Figure 3B). The result was verified by quantitating the intensities of the oligoprecipitated STAT1 bands that were normalized against total amount of input STAT1 (detected with anti-HA antibody) or against the amount of Tyr701 phosphorylated STAT1 (detected with anti-pSTAT1 antibody) (Additional file 3: Figure S3B, C, D and E). Furthermore, overexpression of SUMO-1 hindered STAT1 WT binding to *Irf-1*-oligo in U3A cells, providing further proof that sumoylation inhibits STAT1 DNA-binding (Additional file 3: Figure S3F). Of note, additional oligoprecipitation experiments were carried out with larger protein amounts that would allow detection of sumoylated STAT1. In this experimental setting, sumoylation was not detected in the DNA-bound fraction of STAT1, while SUMO-1 conjugation was readily observed in equal amount of cellular STAT1 (Additional file 3: Figure S3G). These results suggest that sumoylated STAT1 does not bind to DNA or that the DNA-binding of sumoylated STAT1 is significantly diminished.

Promoter bound STAT1 dimer is known to interact with histone acetyl transferase CREB-binding protein (CBP) and acetylation of histones is essential for STAT1-mediated transcriptional activation [26]. Next, we wanted to investigate whether the enhanced DNA-binding of sumoylation deficient STAT1 has functional consequences at the promoter level. To this end, we performed chromatin immunoprecipitation (ChIP) assays on the STAT1 target gene *Gbp-1*. U3A cells stably overexpressing STAT1 WT or sumoylation deficient STAT1 mutant were either left unstimulated or stimulated with human IFN-γ. Immunoprecipitation of cross-linked and scattered chromatin was performed with anti-acetylated histone H4 antibody or anti-rabbit IgG antibody as a control. STAT1 K703R expressing cells showed increased acetylation of histone H4 when compared to STAT1 WT (Figure 3C). This result suggests that the enhanced promoter binding of sumoylation defective STAT1 results in enhanced association of histone acetyl transferases to the promoter leading to increased histone H4 acetylation.

### Sumoylation does not prevent STAT1 dimerization

**Figure 4:**
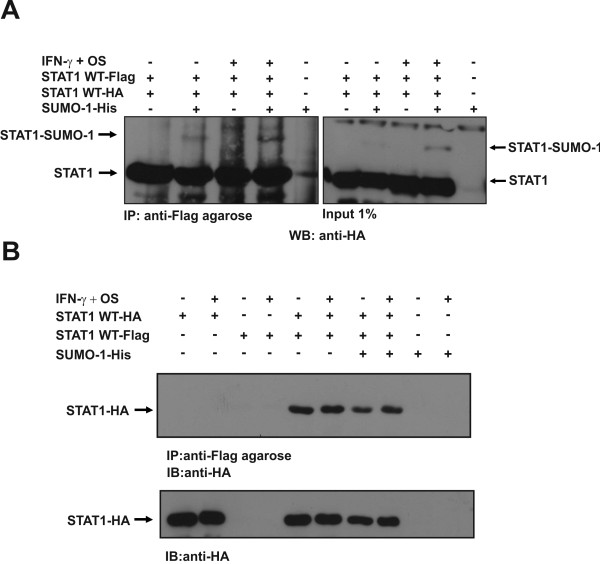
**Sumoylation does not prevent dimerization of STAT1.** (**A**) Sumoylated STAT1 can participate in STAT1 dimers. Cos-7 cells were transiently transfected with STAT1-Flag, STAT1-HA and SUMO-1-His as indicated. 48 hours after transfection cells were either treated or left untreated with 100 ng/ml of human IFN-γ for 45 min and with 0.3 M sorbitol for osmotic shock for 15 min followed by cell lysis. Equal amounts of cell lysates were subjected to co-immunoprecipitation with anti-Flag agarose beads followed immunodetection with anti-HA antibody to reveal STAT1-Flag interacting STAT1-HA proteins. Equal amounts of whole cell lysates (1% input) were separated on the same SDS-PAGE gel and immunoblotted with anti-HA (right panel). Longer exposure time was needed to reveal STAT1-sumoylation bands in the inputs. (**B**) Control experiment showing that anti-Flag agarose does not bind directly to HA-tagged STAT1. Cos-7 cells were transfected, stimulated and immunoprecipitated as described above. IB: immunoblot, IP: immunoprecipitation, OS: osmotic shock.

Dimerization mediated through the interactions between the Tyr701 phosphorylated tail segment of one STAT1 and the SH2 domain of an adjacent STAT1 is considered to be essential for proper DNA-binding and transcriptional activity of STAT1 [3]. To investigate if sumoylation affects STAT1 dimerization and inhibits downstream DNA-binding in this manner, cells were co-transfected with HA- and Flag-tagged STAT1 together or without His-tagged SUMO-1. After 48 hours, the cells were left unstimulated or stimulated with IFN-γ and osmotic shock prior to cell lysis. Equal amounts of whole cell lysates were immunoprecipitated with anti-Flag agarose beads and immunoblotting with anti-HA antibody was used to detect if HA-tagged STAT1 molecules interact with Flag-tagged STAT1. As shown in Figure 4A, a slower migrating band (lanes 2 and 4) corresponding to the size of SUMO-1 conjugated STAT1 was detected with anti-HA antibody, suggesting that sumoylated HA-tagged STAT1 interacts with STAT1-Flag. Figure 4B shows as a control that anti-Flag agarose does not pull down HA-tagged STAT1. These results suggest that sumoylation of STAT1 does not prevent STAT1 dimerization and are consistent with the results that conjugated SUMO moiety affects the interaction between STAT1 and DNA through steric hindrance.

## Discussion

Sumoylation is a common post-translational modification of transcription factors, but in several proteins the physiological functions and molecular mechanisms of this modification have remained enigmatic. Several lines of evidence support the concept that SUMO serves as a negative regulator of STAT1 [10,11,13,14]. Furthermore, the results demonstrating that sumoylation also negatively regulates STAT5-mediated signaling and the only STAT transcription factor in *Drosophila melanogaster*, Stat92E, indicates that sumoylation is an evolutionary conserved post-translational modification for some STAT transcription factors [18,19].

Sumoylation is a highly reversible covalent modification that is regulated through conjugating and deconjugating enzymes. Several studies support the importance of PIAS1-mediated sumoylation of the proteins. Recently, it was shown that PIAS1 regulates oncogenic signaling by sumoylating promyelocytic tumor suppressor (PML) that leads to its ubiquitination and proteosomal degradation [27]. PIAS1 has also been shown to associate with protein-tyrosine phosphatase 1B (PTP1B) and to catalyze its sumoylation, which resulted in down-regulation of the phosphatase activity and inhibited dephosphorylation of the insulin receptor. PIAS1-mediated negative regulation of PTP1B was reversed by SENP1, an isopeptidase that was also shown to regulate sumoylation of STAT5 [18,28]. *Senp1* knock out mice were found to have severe defects in early T and B cell development. The defect in lymphoid development was likely caused by enhanced level of STAT5 sumoylation that subsequently led to decreased STAT5 transcriptional activity [18]. In our experiments removal of conjugated SUMO-1 by SENP1 increased STAT1-mediated reporter gene expression, thus confirming the negative regulatory role of sumoylation for STAT1 (Figure 1B).

STAT1 homodimerization is required for the optimal IFN-γ-mediated gene activation and STAT1 homodimers form a nutcracker-like structure that binds to DNA. The monomers are held together by interface between Tyr701 phosphorylated C-terminal tail segment of one monomer and the SH2 domain of the other [3]. The Lys703 is located adjacent to the dimerization interface and this prompted us to investigate if sumoylation of the Lys703 could affect dimerization or DNA-binding of STAT1. Analysis of the SUMO conjugation consensus site in STAT1 dimer revealed that side chain of Lys703 formed a projection towards DNA. Both Lys703 and Glu705 residues have hydrophilic side chains, which are converted away from the hydrophobic core of SH2 interface. Additionally, the β-sheet structure between two C-tail segments of STAT1 dimer is not likely to be affected by Lys703 mutation to Arg, while this mutation will interrupt the formation of covalent bond with SUMO. The structural analysis revealed that side chain of Lys703 has an interaction phase with Glu632 residue in the SH2 domain of the adjacent monomer. Most probably this interface prevents rotation of this flexible side chain and keeps orientation favorable for the SUMO conjugation. The finding of controlled position of Lys703 also supports the importance of Lys703 as an SUMO acceptor site (Additional file 2: Figure S2). Sumoylation has been shown to impede Tyr701 phosphorylation of STAT1 and subsequent SH2 domain-phospho-Tyr701-mediated homodimerization, leading to formation of semi-phosphorylated dimers that interact through their N-terminal domains [15-17]. Our experimental data indicated that sumoylated STAT1 can form dimers, but it remains to be determined if the interaction is mediated through their N-terminal domains or through the SH2 domains (Figure 4).

To predict how SUMO-1 would structurally orientate in SH2 domain-phospho-Tyr701 interaction-mediated STAT1 dimers, we reconstructed the structure of the disordered loop 684–699 of STAT1, and made a molecular model of sumoylated STAT1 dimer using x-ray structure of TDG-SUMO-1 as a template [25]. This model suggests that the position of SUMO under the loop structure is directed towards DNA and can inhibit interaction with nucleic acids (Figure 2C). Furthermore, results from the oligonucleotide pull down experiments indicated that sumoylation interferes STAT1 binding to STAT1-responsive promoters, as sumoylation deficient STAT1 E705Q showed increased DNA-binding to both *Gbp-1*- and *Irf-1*-oligos (Figure 3A and B), and as SUMO-1 overexpression hindered STAT1 binding to *Irf-1*-oligo (Additional file 3: Figure 3F). The difference in the DNA-binding properties between STAT1 WT and E705Q mutant was not caused by altered Tyr701 phosphorylation (Figure 3A and B) [15-17]. In addition, another sumoylation deficient STAT1 mutant K703R also showed increased binding to *Gbp-1* oligo (data not shown). Taken together, the oligoprecipation experiments are supporting the molecular model where SUMO moiety interferes with DNA-binding of STAT1 (Figure 3A and B, Additional file 3: Figure S3F).

Sumoylated STAT1 was not detected in our oligoprecipitation experiments (Additional file 3: Figure S3G) and this result is consistent with results by Song *et al*. (2006), showing that sumoylated STAT1 does not bind to DNA, or that the bound fraction is very small [29]. In their EMSA studies Song *et al*. also found that sumoylation deficient STAT1 K703R mutant shows prolonged binding to GAS-probe, but unexpectedly sumoylation deficient E705A mutant had similar DNA-binding profile than STAT1 WT [29]. We chose to use STAT1 E705Q mutant in the DNA-binding experiments because the mutant has been reported to have minimal SUMO-independent effects on STAT1 when compared to K703R and E705A mutations [17]. Our results with STAT1 E705Q suggest that sumoylation inhibits DNA-binding properties of STAT1. Supporting this and previously published results of Song *et al.* (2006), we observed that STAT1 K703R has enhanced binding to *Gbp-1*-oligo when compared to STAT1 WT as well (data not shown). Furthermore, sumoylation deficient STAT1 showed enhanced histone H4 acetylation on *Gbp-1* promoter (Figure 3C), thus functionally confirming the enhanced STAT1 promoter binding. Whether sumoylation also alters the interaction with histone acetyl transferases, such as CBP, remains to be determined.

It has become evident that sumoylation participates in regulation of STATs and the precise molecular mechanisms and physiological functions are gradually being revealed. Several studies have analysed sumoylation in STAT1, and sumoylation has been shown to inhibit STAT1 activity by different mechanisms. SUMO conjugation to Lys703 inhibits phosphorylation of Tyr701 [15,16] and prevents paracrystal formation, thereby increasing solubility of STAT1 which subjects STAT1 for dephosphorylation [14,17]. Our results suggest an additional regulatory mechanism for sumoylation and indicate that SUMO moiety is directed towards DNA and can inhibit DNA-binding of STAT1.

## Conclusions

SUMO conjugation to STAT1 has been shown to negatively regulate STAT1-mediated gene responses [11,13,14]. This study was aimed to investigate further the mechanism by which sumoylation regulates STAT1. The inhibitory role of SUMO-1 on STAT1 was confirmed by showing that overexpression of desumoylating enzyme SENP1 increases STAT1-mediated transcriptional activity. A molecular model of sumoylated STAT1 dimer suggested that SUMO-1 is directed towards DNA creating steric hindrance that is able to affect DNA-binding properties of STAT1. Oligoprecipitation experiments were consistent with this model and showed that sumoylation deficient STAT1 mutant has enhanced binding to two independent STAT1 target gene promoters. The difference in DNA-binding was not attributed to the level of Tyr701 phosphorylation of STAT1. Consequently, sumoylation defective STAT1 mutant displayed increased histone H4 acetylation of *Gbp-1* promoter. Taken together, these findings suggest that sumoylation functions as a negative regulator of STAT1 responses by modulating the DNA-binding properties of STAT1.

## Abbreviations

Arg: Arginine; CBP: CREB-binding protein; ChIP: Chromatin immunoprecipitation; DMEM: Dulbecco’s modified eagle’s medium; EDTA: Ethylene diamine tetraacetic acid; EGTA: Ethylene glycol tetraacetic acid; FBS: Fetal bovine serum; GAS: Gamma-activated sequence; GBP: Guanylate binding protein; Gln: Glutamine; Glu: Glutamate; IFN: Interferon; Ile: Isoleucine; IRF: Interferon regulatory factor; L-PEI: Linear-polyethylenimine; Lys: Lysine; NEM: N-ethylmaleimide; PIAS: Protein inhibitor of activated STAT; SDS-PAGE: Sodium dodecylsulfate polyacrylamide gel electrophoresis; Ser: Serine; STAT1: Signal transducer and activator of transcription 1; SUMO: Small ubiquitin-like modifier; Thr: Threonine; Tyr: Tyrosine; Val: Valine.

## Competing interests

The authors declare that they have no competing interests.

## Authors’ contributions

JG carried out STAT1 dimerization experiments and oligoprecipitation experiments, participated in luciferase assay experiments, in the result analysis, study design and drafted the manuscript. SV carried out ChIP experiments, participated in oligoprecipitation experiments, study design, analysis of molecular modeling data and manuscript writing. DU carried out STAT1 desumoylation experiments and participated in luciferase assay experiments, study design and helped to draft the manuscript. JVä, TL and JVa conducted protein modeling and analyzed the constructed models. TL and JVa also took part in manuscript writing. OS participated in study design and coordination and helped to draft the manuscript. All authors read and approved the final manuscript.

## Supplementary Material

Additional file 1 **Stick presentation of antiparallel β-sheet structure of the STAT1 dimer interface.** C-terminal tails of adjacent monomers in STAT1 dimer interface and other residues forming interactions with C-tail residues are coloured with light yellow (monomer 1) and salmon red (monomer 2).Glu705 is coloured with blue and Lys703 with red. β-sheet hydrogen bonding formed between C-terminal tails of monomers and pTyr701 interactions with adjacent SH2 domain are indicated with dashed lines. DNA has been represented as a stick model under the monomer interface and coloured with light blue. Click here for file

Additional file 2 **Orientation of Lys703Arg and Glu705Gln mutated amino acids in STAT1 structure.** Details of the residues 701–705 in the C-terminal tail segment and SUMO consensus site of one monomer are shown in stick (backbone) and stick and ball (side chains) representation (green colour). Adjacent SH2 domain is showed in ribbon, except the interaction forming side chain of Glu632 is shown in stick and ball representation (yellow colour). Side chains of mutated Gln705 and Arg703 (purple colour) are shown together with side chains of WT Glu705 and Lys703. Labels have the same colour as the residues they indicate. Thr704 and Glu632 residues are also indicated with arrows. Hydrogen atoms (H) are coloured in light grey, oxygen atoms (O) in red and nitrogen atoms (N) in blue. Click here for file

Additional file 3 **Analysis of the Tyr701 phosphorylation of the sumoylation deficient STAT1 mutants and the effect of sumoylation on STAT1 DNA-binding.** (A) Tyr701 phosphorylation of WT STAT1 and STAT1 mutants in response to IFN-γ. U3A cells were transfected with STAT1 WT, K703R, E705A, E705Q or Y701F together with SUMO-1-His as indicated. Cells were either left unstimulated or stimulated with IFN-γ prior to lysis. Equal amounts of protein lysates were separated in SDS-PAGE and immunoblotted with anti-pSTAT1 and anti-HA antibodies. (B, C) Comparison of band intensities of the *Gbp-1*-oligoprecipitated WT STAT1 and STAT1 E705Q stained with anti-pSTAT1 (B) and anti-HA (C) antibodies. (D, E) Comparison of WT STAT1 and STAT1 E705Q band intensities from to *Irf-1*-oligoprecipitates stained with anti-pSTAT1 (D) and anti-HA (E) antibodies. Band intensities were studied with ImageJ software and analysed after background subtraction. The intensity of WT STAT1 was set as one in every bar graph (B-E). The error bars indicate SD from two independent experiments. (F) Overexpression of SUMO-1 inhibits STAT1 DNA-binding. U3A cells were transfected with STAT1-HA together with empty vector or SUMO-1-His. Cells were stimulated with IFN-γ and osmotic shock as indicated. After cell lysis equal amounts of protein from the whole cell lysates were oligoprecipitated with *Irf-1*-oligo and separated in SDS-PAGE followed by immunoblotting with anti-pSTAT1 antibody (upper panel). For detection of the total amount of Tyr701 phosphorylated STAT1 and the STAT1 expression levels in the lysates, equal amounts of protein lysates were immunoblotted with anti-pSTAT1 and anti-HA antibodies, respectively (middle and the lower panel). (G) Sumoylated STAT1 does not bind DNA. Cos-7 cells were transfected with WT STAT1-HA together with SUMO-1-His. Prior to lysis cells were stimulated with IFN-γ and osmotic shock as indicated. *Gbp-1*-oligoprecipitated proteins were separated in the SDS-PAGE together with protein samples from the whole cell lysates (10% input). STAT1 and STAT1-SUMO-1 bands were detected by immunoblotting with anti-HA antibody. Both panels are from the same gel, but additional lanes have been cut off from the middle. IB: immunoblot, OP: oligoprecipitation, u.s.: unspecific band. Click here for file
