# Supplementary figures and images for: Structure-function analysis indicates that sumoylation modulates DNA-binding activity of STAT1
Source: BMC Biochem. 2012 Oct 8;13:20. doi: 10.1186/1471-2091-13-20 (PMC3532225; doi:10.1186/1471-2091-13-20)

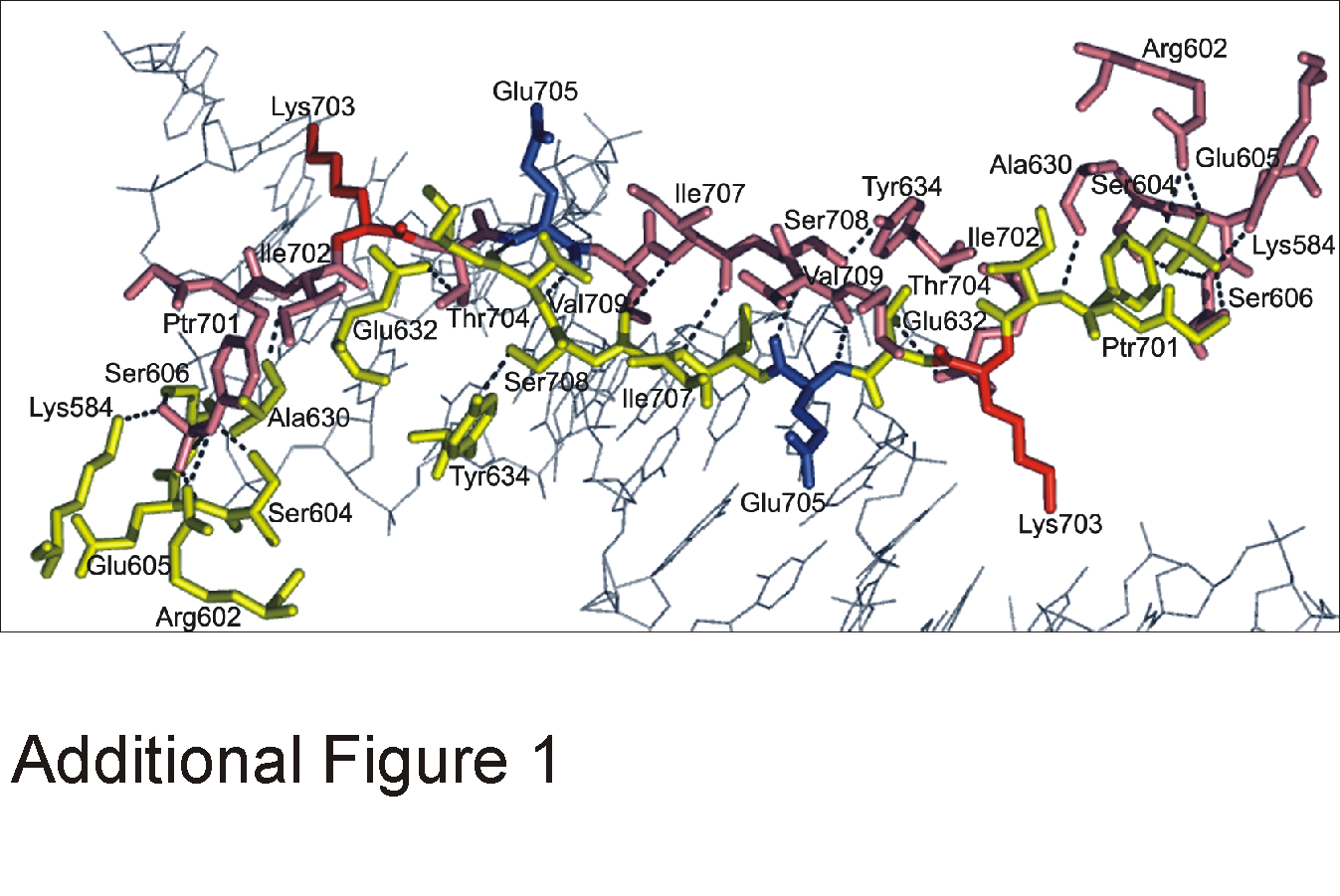

Supplement: Additional file 1 — Stick presentation of antiparallel β-sheet structure of the STAT1 dimer interface. C-terminal tails of adjacent monomers in STAT1 dimer interface and other residues forming interactions with C-tail residues are coloured with light yellow (monomer 1) and salmon red (monomer 2).Glu705 is coloured with blue and Lys703 with red. β-sheet hydrogen bonding formed between C-terminal tails of monomers and pTyr701 interactions with adjacent SH2 domain are indicated with dashed lines. DNA has been represented as a stick model under the monomer interface and coloured with light blue. [file 1471-2091-13-20-S1.png]

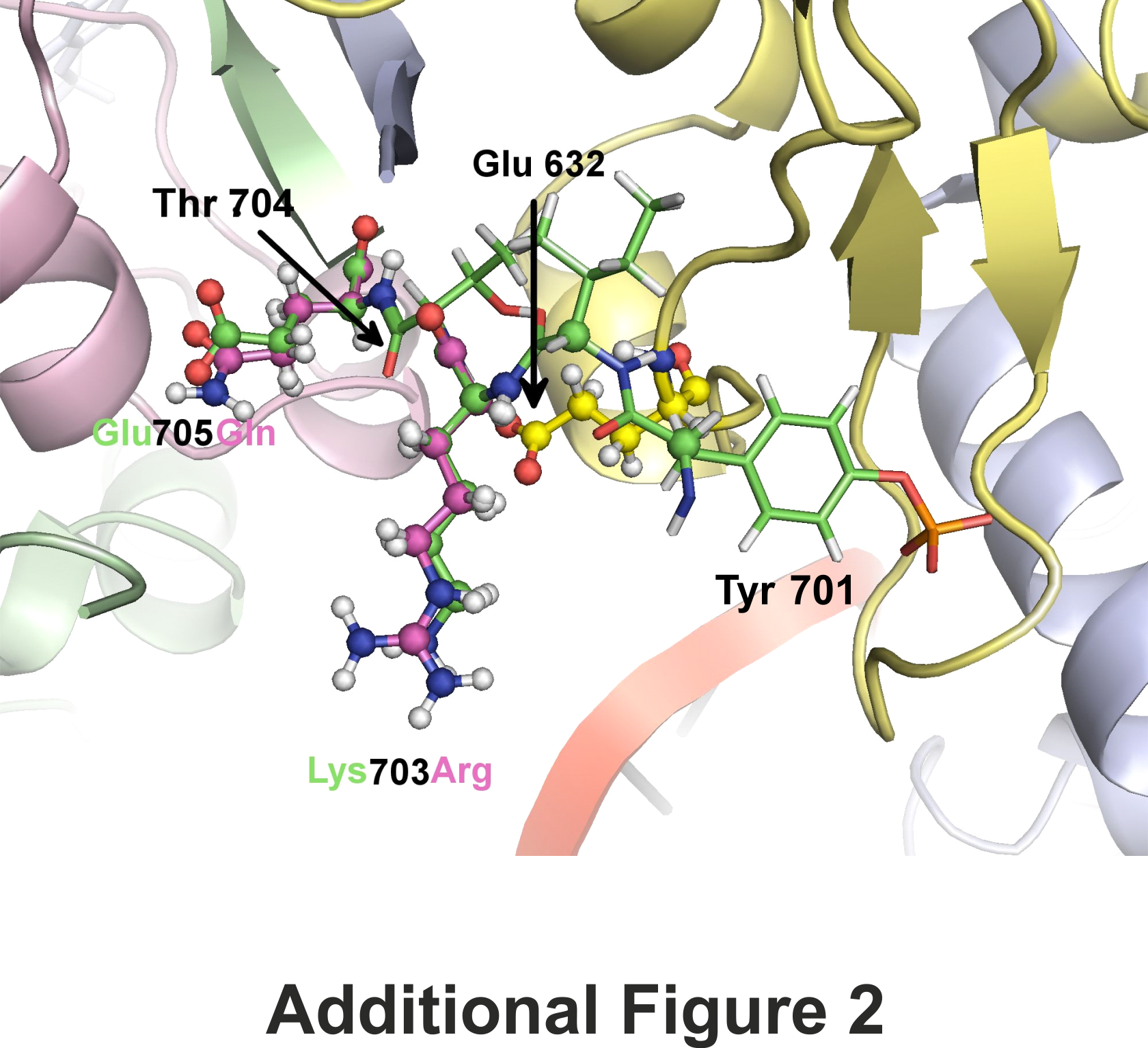

Supplement: Additional file 2 — Orientation of Lys703Arg and Glu705Gln mutated amino acids in STAT1 structure. Details of the residues 701–705 in the C-terminal tail segment and SUMO consensus site of one monomer are shown in stick (backbone) and stick and ball (side chains) representation (green colour). Adjacent SH2 domain is showed in ribbon, except the interaction forming side chain of Glu632 is shown in stick and ball representation (yellow colour). Side chains of mutated Gln705 and Arg703 (purple colour) are shown together with side chains of WT Glu705 and Lys703. Labels have the same colour as the residues they indicate. Thr704 and Glu632 residues are also indicated with arrows. Hydrogen atoms (H) are coloured in light grey, oxygen atoms (O) in red and nitrogen atoms (N) in blue. [file 1471-2091-13-20-S2.png]

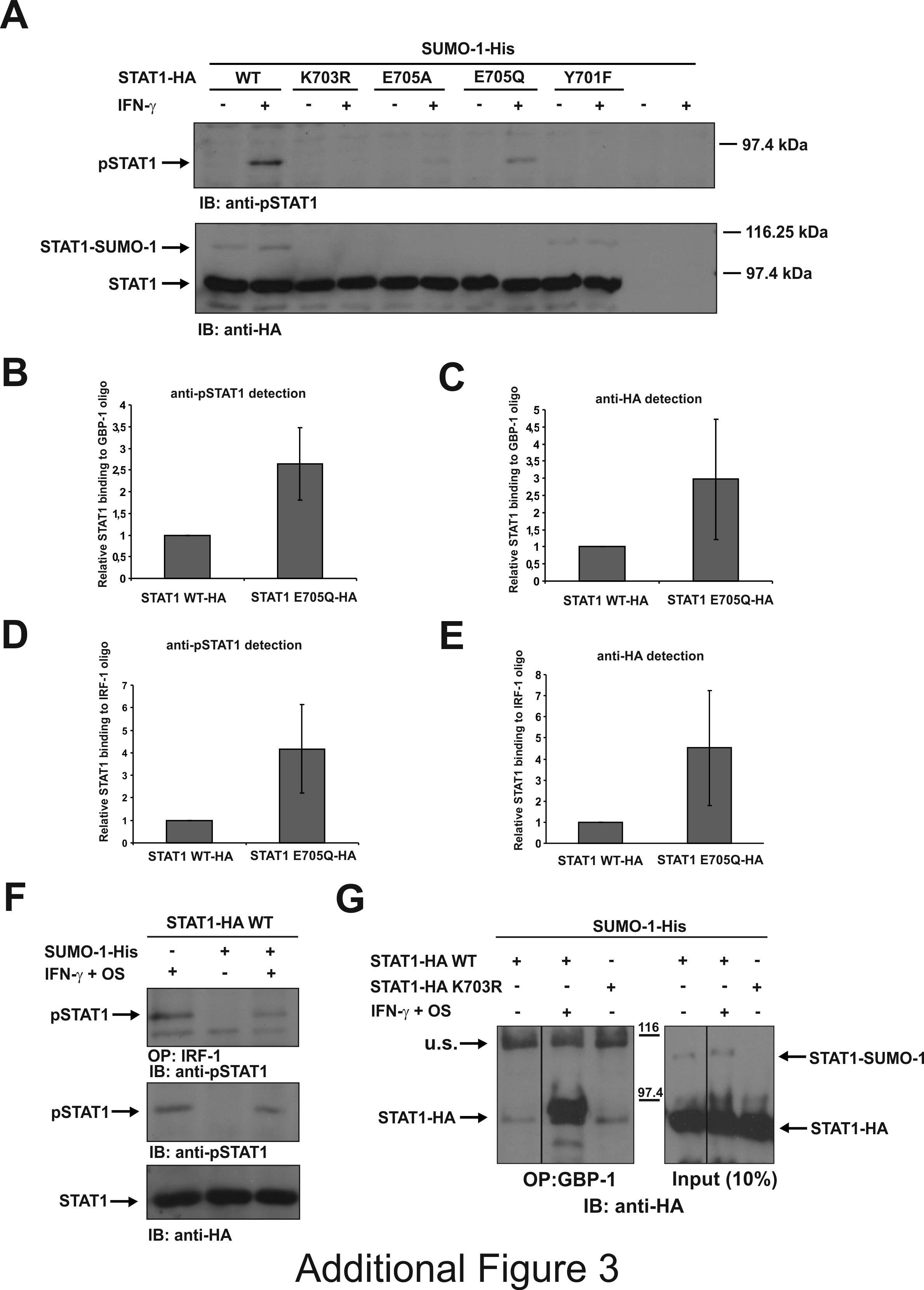

Supplement: Additional file 3 — Analysis of the Tyr701 phosphorylation of the sumoylation deficient STAT1 mutants and the effect of sumoylation on STAT1 DNA-binding. (A) Tyr701 phosphorylation of WT STAT1 and STAT1 mutants in response to IFN-γ. U3A cells were transfected with STAT1 WT, K703R, E705A, E705Q or Y701F together with SUMO-1-His as indicated. Cells were either left unstimulated or stimulated with IFN-γ prior to lysis. Equal amounts of protein lysates were separated in SDS-PAGE and immunoblotted with anti-pSTAT1 and anti-HA antibodies. (B, C) Comparison of band intensities of the Gbp-1-oligoprecipitated WT STAT1 and STAT1 E705Q stained with anti-pSTAT1 (B) and anti-HA (C) antibodies. (D, E) Comparison of WT STAT1 and STAT1 E705Q band intensities from to Irf-1-oligoprecipitates stained with anti-pSTAT1 (D) and anti-HA (E) antibodies. Band intensities were studied with ImageJ software and analysed after background subtraction. The intensity of WT STAT1 was set as one in every bar graph (B-E). The error bars indicate SD from two independent experiments. (F) Overexpression of SUMO-1 inhibits STAT1 DNA-binding. U3A cells were transfected with STAT1-HA together with empty vector or SUMO-1-His. Cells were stimulated with IFN-γ and osmotic shock as indicated. After cell lysis equal amounts of protein from the whole cell lysates were oligoprecipitated with Irf-1-oligo and separated in SDS-PAGE followed by immunoblotting with anti-pSTAT1 antibody (upper panel). For detection of the total amount of Tyr701 phosphorylated STAT1 and the STAT1 expression levels in the lysates, equal amounts of protein lysates were immunoblotted with anti-pSTAT1 and anti-HA antibodies, respectively (middle and the lower panel). (G) Sumoylated STAT1 does not bind DNA. Cos-7 cells were transfected with WT STAT1-HA together with SUMO-1-His. Prior to lysis cells were stimulated with IFN-γ and osmotic shock as indicated. Gbp-1-oligoprecipitated proteins were separated in the SDS-PAGE together with protein samples from the who [file 1471-2091-13-20-S3.png]
